# Epidemiological and clinical characteristics of burns in adults: a 6-year retrospective study in a major burn center in Suzhou, China

**DOI:** 10.3389/fpubh.2024.1413986

**Published:** 2024-06-26

**Authors:** Yong Zhang, Jiandong Su, Yunfeng Liu, Ran Sun, Ruizhu Sun

**Affiliations:** ^1^Department of Burns and Plastic Surgery, The Affiliated Suzhou Hospital of Nanjing Medical University, Suzhou Municipal Hospital, Gusu School, Nanjing Medical University, Suzhou, China; ^2^Department of Ophthalmology, The Affiliated Suzhou Hospital of Nanjing Medical University, Suzhou Municipal Hospital, Gusu School, Nanjing Medical University, Suzhou, China

**Keywords:** burns, burn severity, epidemiology, etiology, outcome

## Abstract

**Background:**

Burns are a prevalent form of unintentional injury and a significant public health concern in developing countries. We aimed to investigate the epidemiological and clinical characteristics of adult burn patients at a major center in Eastern China.

**Methods:**

This 6-year retrospective study analyzed patients with varying degrees of burns between January 2017 and December 2022 at the Suzhou Burns and Trauma Center. The study extracted demographic, clinical, and epidemiological data from electronic medical records for analysis.

**Results:**

The study included 3,258 adult patients, of which 64.3% were male. The largest age group affected 30–59-year-old adults (63.04%). Scalds were the leading cause of burns (1,346, 41.31%), followed by flames (1,271, 39.01%). The majority of burn hospitalizations were those with moderate burns (1791, 54.97%). The morbidity rate was low at 0.68%, while mortality was strongly associated with age, etiology, and total body surface area. Patients with certain types of burns, such as explosions, hot crush injuries, and electric burns had more operations, longer lengths of hospital stay, and higher costs compared to those with scalds and flame injuries.

**Conclusion:**

Different prevention strategies should be formulated according to different etiologies, ages, and genders.

## Introduction

Burns are a significant public health issue in developing countries and one of the prevalent unintentional injuries in daily life. With improvements in medical care and the continued standardization and availability of clinical treatment, there has been a decrease in burn incidence, burn mortality, burn severity, and lengths of hospital stay globally (1, 2). However, the mortality rate from burns remains high worldwide, particularly in developing countries. The World Health Organization estimates that approximately 180,000 people die from burns annually, with a significantly higher prevalence in developing countries (3). Burns can cause significant harm to the skin and other organs, resulting in death, disability, serious emotional and psychological complications, and increased financial burdens for families (4, 5). Burn victims require not only initial treatment in the acute phase but also subsequent reconstruction, rehabilitation, and long-term anti-scarring treatment (6, 7). Burns and their sequelae therefore have a serious negative impact on the patients, their families, and society. Specific and timely epidemiological investigations are essential in developing effective prevention strategies, reducing accidents, improving treatment outcomes, and reducing the economic burden (8).

Our burn center is located in Suzhou City, Jiangsu Province, with a resident population of 12.96 million, the highest in the province. Suzhou is located in the central part of the Yangtze River Delta and the southeastern part of Jiangsu Province, between longitude 119°55′–121°20′E and latitude 30°47′–32°02′N, with a subtropical monsoon maritime climate. Burn patients mainly suffer from scald, fire, electricity, and liquefied petroleum gas burns related to residential life and occupational-related burns in industrial sites. Suzhou Burns and Trauma Center is a provincial regional treatment center and the largest burn center in the Suzhou area. The center treats burn and scald, bulk burns, extra severe burns, respiratory burns, trauma repair, keloid surgery, and burns rehabilitation in Suzhou and the surrounding areas. It has 80 beds and 8 Burn Intensive Care Unit beds, an annual outpatient clinic volume of 30,000, and inpatient hospitalization of more than 1,000 times.

There is a lack of reports on burn epidemiology in the Suzhou region. In this study, we aimed to investigate the epidemiological and clinical characteristics of adult burn patients in Suzhou. We retrospectively analyzed information on the treatment of patients hospitalized in our burn center during the period 2017–2022. We analyzed the epidemiological characteristics, etiology, and outcomes of burn patients in our hospital over the last 6 years. This may provide useful epidemiologic information for the development of burn prevention strategies.

## Methods

### Study design and data collection

The study collected medical records of 3,258 adult burn patients aged 18 years and older with complete data who were admitted to the burn center of The Suzhou Hospital Affiliated to Nanjing University between January 2017 and December 2022. Before analyzing the data and writing the manuscript, we anonymized all patients’ information. The inclusion and exclusion criteria were strictly followed for the selection of patients.

Inclusion Criteria: (1) Age ≥ 18 years. (2) Inpatients in the burn center of our hospital from January 2017 to December 2022. (3) The primary diagnosis is burns. (4) Complete medical records. Exclusion criteria: (1) Burns are not the main diagnosis. (2) The patient was initially diagnosed with a burn, but was readmitted to the hospital due to burn sequelae. (3) Patients diagnosed with burns but have only been treated in an emergency or outpatient setting. (4) Patients with incomplete epidemiological data retention.

The electronic medical records of the hospital were used to extract data on age, gender, admission and discharge dates, burn injury etiology, depth and extent of burns, burn severity, injured anatomic locations, surgery status and frequency, patient outcomes, length of stay in hospital (LOS), and medical costs. The study encompassed seven different etiologies, including scald, flame, chemical, explosion, hot crush injury, electricity, and others. This study classified patients into three age groups: 18–29 years old, 30–59 years old, and ≥ 60 years old. Data collection and entry were performed by a single investigator, with accuracy checked by another researcher. In cases of suspected errors, a third researcher participates in proofreading, and the team collaborates to rectify any discrepancies.

The severity of the burn area was evaluated using the Chinese rule-of-nine and rule-of-palms (9). Mild burns are defined as the second-degree burns area is less than 10%. Moderate burns are defined as the second-degree burns area between 11 and 30%, or the third-degree burns area is less than 10%. Severe burns are defined as the total area is between 31 and 50%, or the third-degree burns area is between 11 and 20%, or the burn area is less than the above, but suffered shock, heavier inhalation injuries, and compound injuries. Extremely severe burns are defined as the total burn area is more than 50%, or the third-degree burns area is more than 20% (10).

Patients’ outcomes were classified below. If a patient died before discharge, we recorded it as “deceased.” If the burn wound was not decreased at discharge, we recorded it as “invalid.” If the burn wound was reduced but not healed, we recorded it as “improved.” If the burn wound had healed above 95% without any secretion, we recorded it as “cured.” The effective rate was calculated by summing the improvement rate and the cure rate.

### Statistical analysis

The data was processed and analyzed using Microsoft Excel (2021, Microsoft Corp., United States) and SPSS (26, IBM Corp., United States). Categorical variables were presented as numbers (percentages). Continuous variables with a non-normal distribution were described as median (interquartile range, IQR). Group comparisons were conducted using non-parametric tests (Mann–Whitney U test or Kruskal-Wallis rank sum test). The study employed Pearson’s correlation coefficient to establish relationships between variables. *p* values of less than 0.05 were considered statistically significant.

## Results

### Demographic characteristics

This study included 3,258 adult inpatients, of whom 2095 (64.30%) were male and 1,163 (35.70%) were female. The median age of patients was 47 years old (IQR, 24), ranging from 18 to 94 years old. The largest age subgroup was the 30–59 years group, which accounted for 63.04% (2054) of the patients, followed by the ≥60 years group (21.39%, 697 patients) and the 18–29 years group (15.56%, 507 patients). Over the 6 years, an average of 543 patients were admitted annually. Since 2018, there has been a gradual decrease in the number of patients admitted each year, with a trend toward a higher median age of patients (Table 1).

**Table 1:** Demographics and clinical characteristics of patient population from 2017 to 2022.

Year	No. of patients	Sex (Male/Female, %)	Age median (IQR), year	Burn severity (n, %)			
				Mild	Moderate	Severe	Extra severe
2017	679	435/244(1.78:1)	46 (24)	227(33.43)	369(54.34)	47(6.92)	36(5.30)
2018	681	436/245(1.78:1)	45 (24)	239(35.10)	373(54.7)	39(5.73)	30(4.41)
2019	670	405/265(1.53:1)	47 (24)	255(38.06)	358(53.43)	37(5.52)	20(2.99)
2020	453	294/159(1.85:1)	47 (24)	136(30.2)	263(58.06)	26(5.74)	28(6.18)
2021	410	265/145(1.83:1)	49 (21)	127(30.98)	230(56.10)	35(8.54)	18(4.39)
2022	365	260/105(2.48:1)	49 (24)	108(29.59)	198(54.25)	26(7.12)	33(9.04)
Total	3,258	2095/1163(1.8:1)	47 (24)	1,092(33.52)	1791(54.97)	210(6.45)	165(5.06)
*P-*value	–	<0.05	<0.05	<0.01			

IQR interquartile range, *p* < 0.05 were considered statistically significant.

### Seasonal distribution

**Figure 1:**
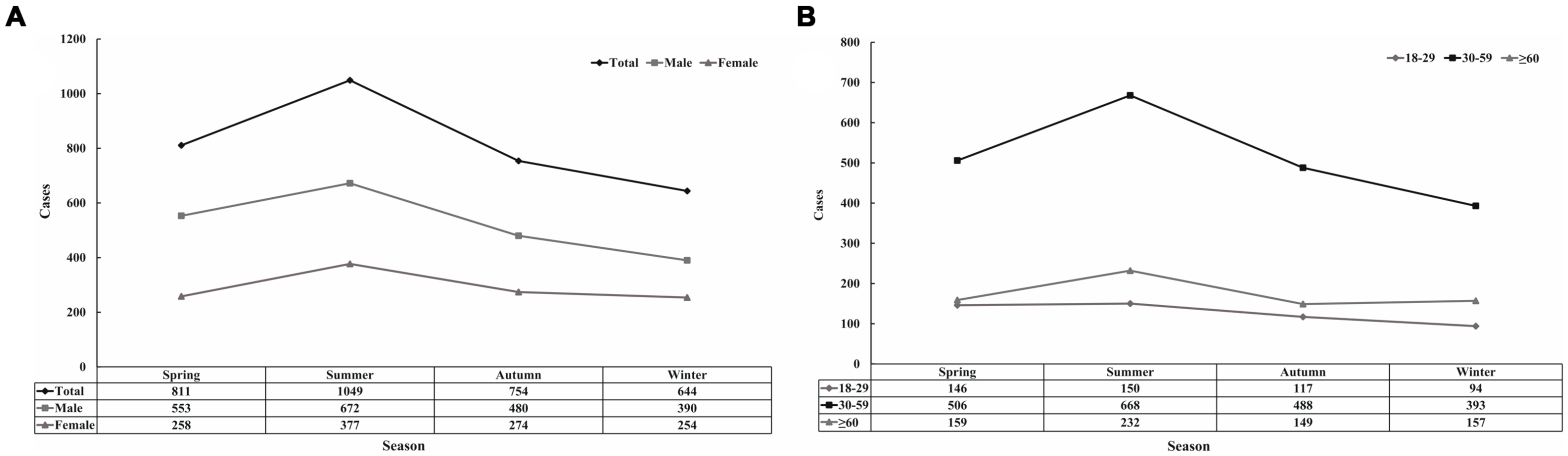
Distribution of burn patients by season. **(A)** Distribution of patients in different gender groups. **(B)** Distribution of patients in different age groups.

The prevalence of burns exhibited seasonal variation. Most of the hospitalizations occurred in summer (32.20%, June to August), followed by spring (24.89%, March to May), autumn (23.14%, September to November), and winter (19.77%, December to February). The seasonal distribution of burns exhibited a similar trend in both males and females (Figure 1A). There were differences in the season of incidence of burns among patients in different age groups (Figure 1B).

### Etiology

**Figure 2:**
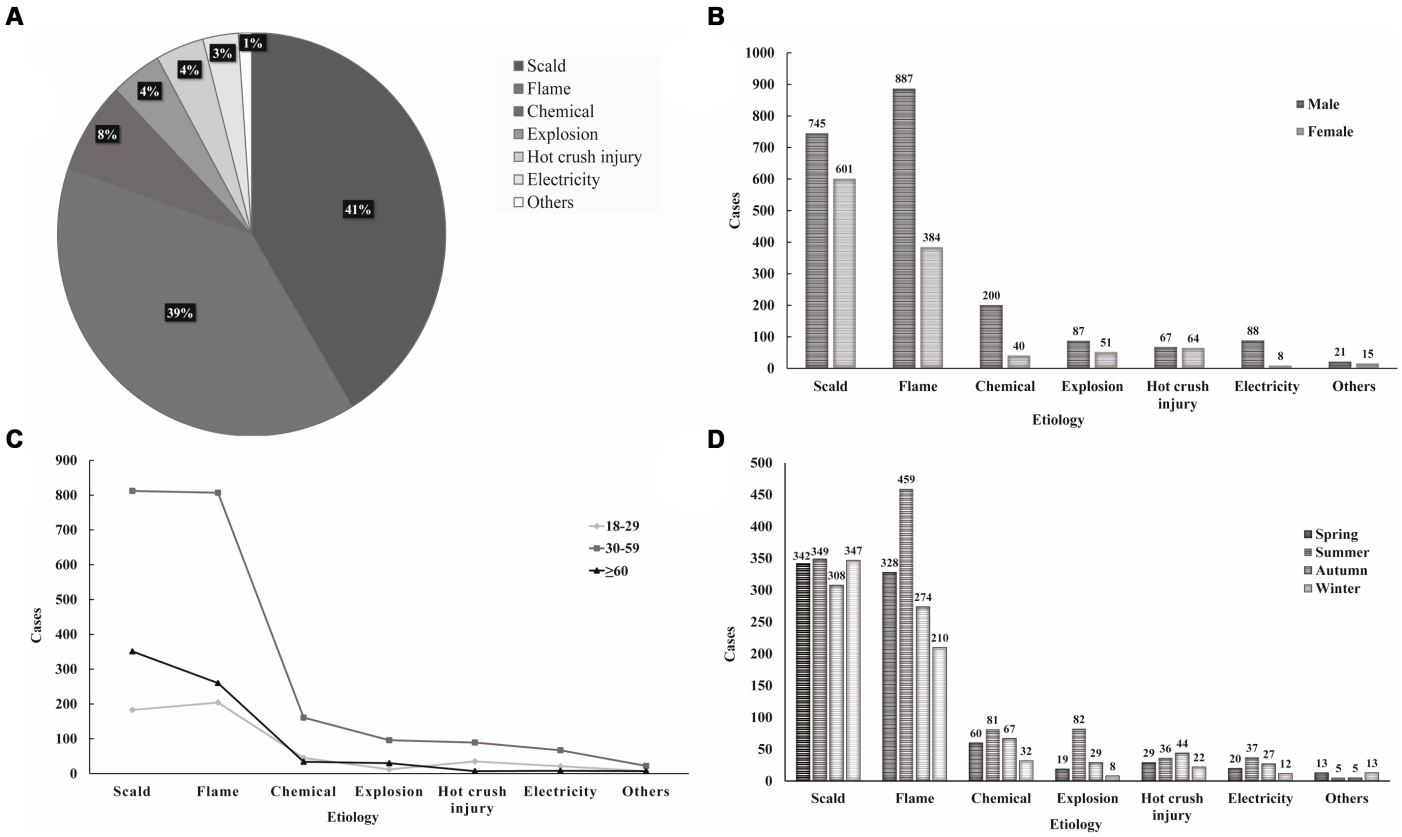
Etiology analysis-proportions and distributions. **(A)** Proportions of different etiologies. **(B)** Distribution of etiology by gender. **(C)** Distribution of etiology by age. **(D)** Distribution of etiology by season.

As shown in Figure 2A, scald (41.31%) and flame (39.01%) injuries were the most common causes in this study. Flame burns were the most prevalent cause in males (42.34%) while scalds were the most common cause in females (51.68%) (Figure 2B). Flames and scalds were the leading causes in all age groups (Figure 2C). The prevalence of flame and explosion peaked during the summer months (Figure 2D).

### Burn sites

This study classified burns into six sites based on common diagnosis (Table 2). In this study, 70.4% of patients had combined limb burns, followed by Face/Neck/Scalp (46.3%) and hands (45.9%). The limbs were the most common site of burns for individuals of different genders and age groups (Table 2). A significant correlation was found between burn site and burn etiology. The most common site of flame injuries was the Face/Neck/Scalp and limbs, while hot crush and electrical injuries were most common on the hands. Explosive injuries often involve multiple burns to the body.

**Table 2:** Distribution of burn sites by age group, gender, and etiology.

	Face/Neck/Scalp n (%)	Trunk n (%)	Limbs n (%)	Hands n (%)	Feet n (%)	Hip and perineum n (%)
Total	1,507 (46.30)	971 (29.80)	2,294 (70.4)	1,497 (45.9)	873 (26.8)	299 (9.2)
Gender						
Male	1,066 (50.88)	656 (31.31)	1,433 (68.4)	1,041 (49.69)	557 (26.59)	190 (9.07)
Female	441 (37.92)	315 (27.09)	861 (74.03)	456 (39.21)	316 (27.17)	109 (9.37)
*P*-value	<0.001	<0.05	<0.001	<0.001	0.718	0.774
Age group						
18–29	223 (43.98)	159 (31.36)	351 (69.23)	243 (47.93)	117 (23.08)	35 (6.9)
30–59	998 (48.59)	639 (31.11)	1,444 (74.03)	967 (47.08)	545 (26.53)	187 (9.1)
≥60	286 (41.03)	173 (24.82)	499 (71.59)	287 (41.18)	211 (30.27)	77 (11.05)
*P* value	<0.001	<0.01	0.664	<0.05	<0.05	<0.05
Etiologies						
Scald	322 (23.92)	374 (27.79)	946 (70.28)	293 (21.77)	426 (31.65)	143 (10.62)
Flame	958 (75.37)	397 (31.24)	975 (76.71)	827 (65.07)	270 (21.24)	89 (7.00)
Chemical	76 (31.67)	59 (24.58)	149 (62.08)	70 (29.17)	61 (25.42)	17 (7.08)
Explosion	137 (99.28)	113 (81.88)	136 (98.55)	126 (91.30)	96 (69.57)	46 (33.33)
Hot crush injury	7 (5.34)	11 (8.4)	46 (35.11)	97 (74.05)	1 (0.76)	1 (0.76)
Electricity	6 (6.25)	16 (16.67)	42 (43.75)	84 (87.5)	19 (19.79)	3 (19.79)
others	1 (2.78)	1 (2.78)	0 (0)	0 (0)	0 (0)	0 (0)
*P-*value	<0.001	<0.001	<0.001	<0.001	<0.001	<0.001

### Burn severity and burn extent

**Figure 3:**
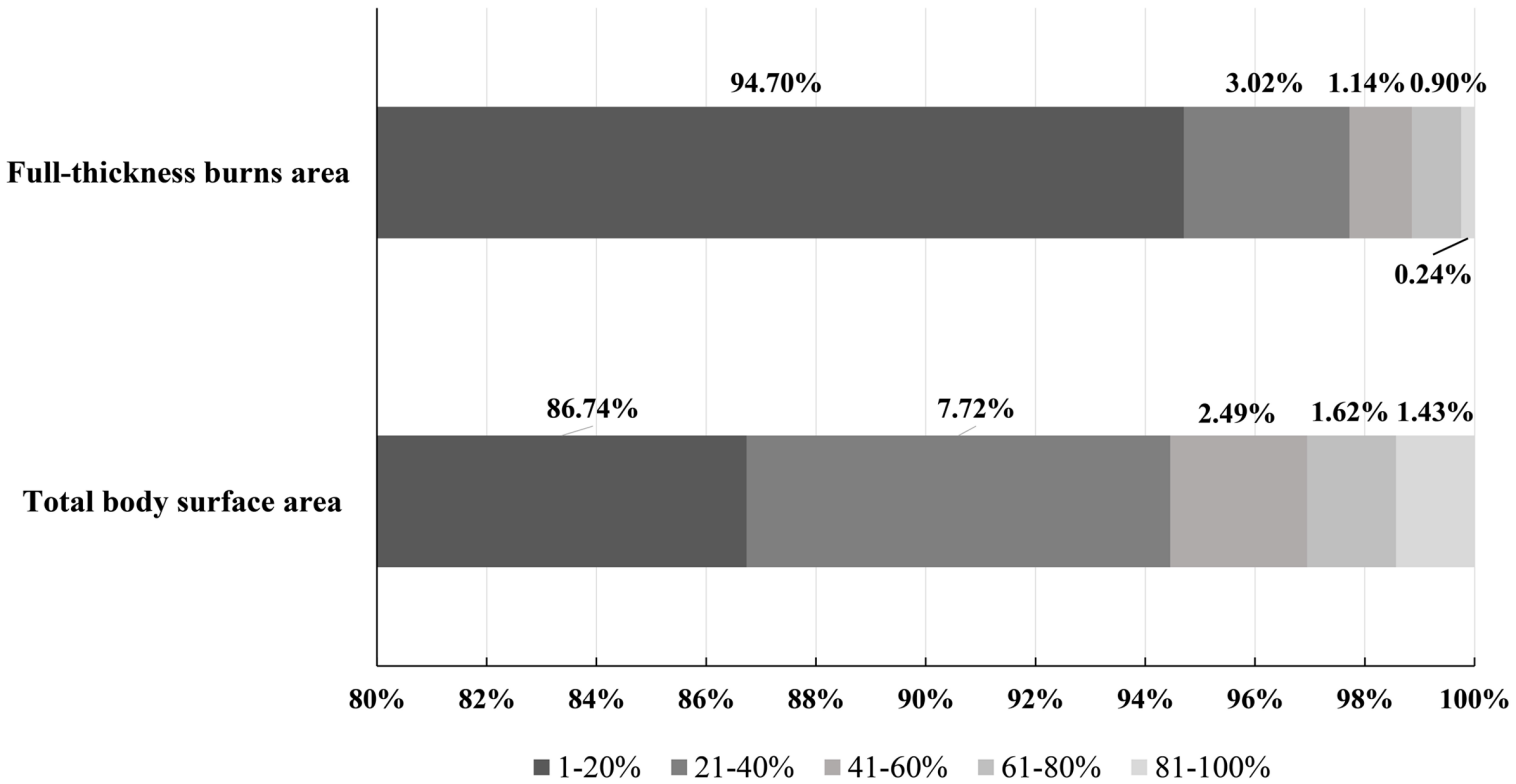
Distribution of total body surface area (TBSA) and full-thickness burns area.

The total burn surface area (TBSA) ranged from 0 to 98%, with a median of 6% (IQR,11%). Thirty four patients in this study had simple inhalation injuries with a TBSA of 0. This study had 1,226(37.63%) patients with full-thickness burns. The area of full-thickness burns ranged from 1 to 90%. Figure 3 shows the distribution of TBSA and full-thickness burns area in all patients. The area of TBSA was larger in males than in females (*p* < 0.05). Significant differences in TBSA were observed between patients with different etiologies (Table 3). In this study, moderate burns (1791, 55%) were most common in hospitalized patients, followed by mild burns (1,092, 35.5%). Explosion injuries were most likely to result in extremely severe burns (84, 69.87%) (Table 3).

**Table 3:** Distributions of total body surface area (TBSA), burn severity by gender, age, etiology.

	TBSA, % median (IQR)	Mild n (%)	Moderate n (%)	Severe n (%)	Extra severe n (%)
Total	6 (11)	1,092 (35.5)	1791 (55.0)	210 (6.4)	165 (5.1)
Gender					
Male	7 (12)	680 (32.46)	1,159 (55.32)	142 (6.78)	114 (5.44)
Female	6 (10)	412 (35.43)	632 (54.34)	68 (5.85)	51 (4.39)
*P-*value	<0.05	0.191			
Age group					
18–29	6 (10)	159 (31.36)	297 (58.58)	33 (6.51)	18 (3.55)
30–59	7 (12)	686 (33.40)	1,135 (55.26)	123 (5.99)	110 (5.36)
≥60	7 (9)	247 (35.33)	359 (51.51)	54 (7.75)	37 (5.31)
*P-*value	0.055	0.15			
Etiologies					
Scald	5 (8)	534 (39.67)	765 (56.84)	30 (2.23)	17 (1.26)
Flame	10 (12)	454 (35.72)	644 (50.67)	114 (8.97)	59 (4.64)
Chemical	4 (6)	73 (30.42)	153 (63.75)	9 (3.75)	5 (2.08)
Explosion	55 (40)	1 (0.72)	5 (3.62)	48 (34.78)	84 (60.87)
Hot crush injury	1 (1)	3 (2.29)	127 (96.95)	1 (0.76)	0 (0)
Electricity	2 (3)	5 (5.21)	86 (89.58)	5 (5.21)	0 (0)
Others	0 (0)	22 (61.11)	11 (30.56)	3 (8.33)	0 (0)
*P*-value	<0.001	<0.001			

### Surgery and outcome

The distribution of surgery is shown in Table 4. Whether to undergo surgical treatment was significantly associated with age, etiology, and TBSA (Table 4). The percentage of patients receiving surgical treatment was lower in the ≥60 years older group (*p* < 0.001). With increasing TBSA, the proportion of patients undergoing surgery increased significantly (*p* < 0.001). The highest percentage of patients who underwent surgery were those with hot crush injuries (79.39%), followed by explosion injuries (71.01%) and electrical injuries (70.83%). Sex, age, etiology, and surgery were significantly correlated with the cure rate. The effective rate was significantly correlated with age, etiology, and TBSA. The study showed a mortality rate of 0.68%, which was strongly associated with age, etiology, and TBSA (Table 4).

**Table 4:** Distributions of surgery, outcome, length of stay (LOS), and cost by gender, age, etiology, total body surface area (TBSA), and full-thickness burns.

	No. of patients	Surgery n (%)	Cure rate n (%)	Effective rate n (%)	Mortality n (%)	LOS (days) median (IQR)	Cost (CNY) median (IQR)
Total	3,258	1,275 (39.13)	2,296(70.47)	3,185 (97.76)	22 (0.68)	14(17)	16,126 (33,408)
Gender							
Male	2095	841 (40.14)	1,502(71.69)	2044 (97.57)	17 (0.81)	15 (18)	17,984 (26,702)
Female	1,163	434 (37.32)	794 (68.27)	1,141(98.11)	4 (0.34)	14 (15)	13,972 (26,702)
*P*-value	–	0.113	<0.05	0.316	0.11	<0.01	<0.001
Age group (year)							
18–29	507	224 (44.18)	373 (73.57)	504 (99.41)	0 (0)	14 (18)	16,414 (35,157)
30–59	2054	832 (40.51)	1,478 (71.96)	2024 (98.54)	8 (0.39)	15 (17)	16,849 (34,268)
≥60	697	219 (31.42)	445 (63.85)	657 (94.26)	13 (1.87)	13 (15)	14,189 (28,424)
*P*-value	–	<0.001	<0.001	<0.001	<0.001	<0.01	<0.01
Etiologies							
Scald	1,346	495 (36.78)	907 (67.38)	1,335 (99.18)	3 (0.22)	13 (13)	12,587 (22,845)
Flame	1,271	375 (29.50)	907 (71.36)	1,232 (96.93)	11 (0.87)	13 (15)	15,121 (32,905)
Chemical	240	134 (55.83)	175 (72.92)	234 (97.50)	2 (0.83)	16 (16)	20,488 (28,412)
Explosion	138	98 (71.01)	110 (79.71)	123 (89.13)	5 (3.62)	39 (36)	200,705 (227,879)
Hot crush injury	131	104 (79.39)	109 (83.21)	130 (99.24)	0 (0)	26 (17)	41,182 (33,631)
Electricity	96	68 (70.83)	68 (70.83)	95 (98.96)	0 (0)	28.5 (32.75)	39,121 (66,840)
others	36	1 (2.78)	20 (55.56)	36 (100)	0 (0)	4 (7.5)	4,090 (8,039)
*P*-value	–	<0.001	<0.001	<0.001	<0.001	<0.01	<0.001
TBSA							
0–29	2,932	1,053(35.91)	2065 (70.43)	2,905 (99.08)	4 (0.14)	13 (14)	13,863 (25,822)
30–49	183	122 (66.67)	133 (72.68)	166 (90.71)	4 (2.19)	27 (25)	88,159 (104,190)
≥50	143	100 (69.93)	98 (68.53)	114 (79.72)	13 (9.09)	47 (41)	302,332 (319,663)
*P*-value	–	<0.001	0.709	<0.001	<0.001	<0.01	<0.001
Full-thickness burns area							
0	2031	788 (38.80)	1,437 (70.75)	1985 (97.74)	11 (0.54)	14 (16)	16,117 (33,572)
1–9	1,063	422 (39.70)	731 (68.77)	1,038 (97.65)	10 (0.94)	15 (17)	16,373 (34,267)
10–19	81	29 (35.80)	62 (76.54)	79 (97.53)	0 (0)	13 (12)	12,872 (21,776)
≥20	83	36 (43.37)	66 (79.52)	83 (100)	0 (0)	15 (17)	16,696 (29,917)
*P*-value	–	0.743	0.1	0.575	0.414	0.747	0.470
Surgery no.							
0	1983	–	1,206 (60.82)	1925 (97.08)	15 (0.76)	10 (6)	9,138 (10,261)
1	786	–	671 (85.50)	778 (98.98)	1 (0.13)	20 (13)	32,123 (33,132)
2	324	–	286 (88.27)	320 (98.77)	3 (0.93)	29 (17.75)	46,845 (46,813)
3 and more	165	–	133 (81.82)	162 (98.18)	2 (1.21)	50 (24)	154,969 (258,156)
*P*-value	–		<0.001	<0.05	0.179	<0.001	<0.001

### Length of stay and hospitalization costs

The median Length of stay (LOS) for all burn patients was 14 days (range 1–165 days). The median hospitalization cost was RMB 16,126 (range 49–314,2,606). Table 4 shows the distribution of LOS and hospitalization costs for all patients. LOS and hospitalization cost were associated with gender, age, etiology, TBSA, and number of surgeries. We performed a person correlation analysis of LOS with age, TBSA, full-thickness burn area, hospital cost, and number of surgeries. As shown in Table 5, we found that age was negatively associated with LOS (*p* < 0.01), while TBSA (*p* < 0.001) and the number of surgeries (*p* < 0.001) were positively associated with LOS. Hospital costs were positively associated with age (*p* < 0.001), TBSA (*p* < 0.001), number of surgeries (*p* < 0.001), and LOS (*p* < 0.001).

**Table 5:** Pearson correlation analysis between LOS and age, total body surface area (TBSA), three-degree burn area, etiology, number of surgeries, length of stay (LOS), and cost.

Variables	LOS		Cost	
	*r*	*P*-value	*r*	*P*-value
Age	−0.057	<0.01	0.604	<0.001
TBSA	0.484	<0.001	0.618	<0.001
Full-thickness burn area	0.008	0.662	−0.013	0.441
Number of surgeries	0.680	<0.001	0.484	<0.001
LOS	–	–	0.604	<0.001
Cost	0.604	<0.001	–	–

## Discussion

This retrospective study analyzed the epidemiological data and clinical outcomes of burn patients aged 18 years and above who were admitted to our burn center during the period 2017–2022. The number of adult burn patients decreased annually over the six-year period, in accordance with global trends in burn incidence (1, 11–13). Suzhou is one of the more economically developed regions in China. In May 2018, the local government initiated the “331 Special Action” within the city of Suzhou. A series of rigorous investigation and rectification measures were implemented in areas prone to fires, including “three-in-one” locations, rental housing, electric bicycles, “nine small places, “and small and micro enterprises. This action has the dual benefit of reducing fire hazards in residents’ lives and work. Since 2019, there has been a notable decline in the number of burn patients requiring hospitalization at our hospital. This observation suggests that burns, as an unintentional injury, can be effectively mitigated through the implementation of appropriate prevention strategies and the enhancement of residents’ awareness regarding fire and electrical safety. The median age of admitted patients exhibited an upward trend in this study. This may be because as society ages and the physically fit of the older adult improves, the retirement age of the older adult is delayed, the older adult are still engaged in jobs and activities that carry a higher risk of burns. Analyses of populations in other regions of the country have also revealed a notable increase in the proportion and the mean age of older adult burn patients compared to previous years (14, 15). The hospitalization rate was higher among male patients. Additionally, the age group most affected by burns was predominantly 30–59-year-old group. This finding is consistent with most previous studies. It is possible that middle-aged men, as the main labor force of the family, are more frequently exposed to hazardous work environments (16, 17). Previous studies have indicated that the seasonal trend in the prevalence of burns among females was not significant (18, 19). However, in this study, both males and females in the present study showed a high prevalence of burns during the summer. China is a vast country with different climates in different regions. Suzhou has a subtropical monsoon maritime climate with high temperatures in summer, which makes it more prone to fires in both the workplace and home life. In the present study, the results also showed that the seasonal distribution of scald injuries did not change significantly, whereas flame injuries were significantly more common in summer. Older adult patients ≥60 years of age had a higher prevalence of burns in winter than the other two age groups. Since Suzhou has no centralized heating in winter and the weather is wet and cold, older adult patients usually need to use hot water bags, electric blankets, or other electrical appliances to keep warm. As the older adult have a poorer sense of temperature, they cannot get away from the heat source in time, thereby increasing their risk of burn injury. Additionally, their slower reaction speed and mobility make them more vulnerable to burns in case of a fire. Scalding and flame injuries were the major causes in this study, which is similar to the results of most previous studies (19, 20). This study found that males were more likely to experience flame burns, while females were more prone to scalding. It may be because females are more frequently engaged in domestic activities, which increases their likelihood of exposed to hot water or soup. Men are comparatively more prone to encountering hazardous work environments. Certain types of burns, such as chemical, hot crush, and electrical injuries, are strongly associated with occupational exposure. These types of burns are more prevalent in male patients (21). Consequently, prevention and promotion strategies should be developed in accordance with the specific characteristics of burns in different populations. For the older adult population and the female group, community publicity should be increased to reinforce the safety education on the use of electrical appliances and kitchen utensils in the daily lives of residents. For middle-aged men, it is imperative to reduce the occurrence of occupational injuries, eliminate safety hazards in industrial sites in a timely manner, and formulate distinct publicity and prevention strategies based on the characteristics of each occupation. The study found that the limbs were the most common site of burns. The Face/Neck/Scalp and hands were also frequently affected. This is similar to previous findings in other organizations (19, 22). Other studies have found that hand (23) and the Face/Neck/Scalp was the most common site of burns (16). Performing important functions in both work and daily life, limbs and hands are often exposed to hazardous heat sources, making them more vulnerable to injury. The skin on the Face/Neck/Scalp is not protected by clothing, making it difficult to avoid burns in the event of fire. Scars caused by burns to the Face/Neck/Scalp burns can affect the patient’s facial appearance and psychological health (24). Scar contractures caused by burns to the limbs and hands can lead to limited joint movement, impeding patients’ ability to engage in work or daily life activities. Active orthopedic repairment and functional exercise are needed during the recovery period of burns (25). Concurrently, clinicians and family members must be aware of the potential psychological changes that burn patients may experience and provide timely psychological counseling.

The highest percentage of patients with burns of varying degrees of severity were those with moderate burns, followed by those with mild burns. Some patients with mild burns were treated and monitored only on an outpatient and emergency clinics. As this study involved only inpatients, the actual percentage of patients with mild burns may be higher than the statistical results. At present, there is no strict international standard for grading burn severity. The burn severity grading standard used in this study is the most prevalent in mainland China. However, there have been fewer previous studies analyzing burn injuries based on burn severity grading in China. Consequently, there is a need for more epidemiologic studies. The present study showed that there were significant differences in the TBSA and burn severity among patients with different etiologies. Chemical injuries, hot crush injuries, and electrical injuries had relatively small TBSA and mostly moderate burns. Explosion injuries usually cause large TBSA resulting in extremely severe and severe burns. This is due to the distinct characteristics of injuries of different etiologies. Explosion injuries in Suzhou occurred mostly during the summer. The high temperature generated by an explosion causes instantaneous burns to any exposed skin. Explosion injuries in Suzhou are predominantly domestic gas explosions and occur mostly in older neighborhoods. This indicates that government departments need to pay attention to the renovation and maintenance of gas pipelines. In addition, government departments should disseminate information to residents on the safe use of household gas equipment to prevent major accidents. Clinician’s attention is often focused on patients with severe and extremely severe burns. It is crucial to recognize that in certain types of burns, although the burn area is small and the depth of the burn is shallow, there is also a threat to the patient’s functional recovery of the injured area, or even pose a threat to life. Although the TBSA area is small and mostly moderate hot crush injuries are complex and deep. Such injuries can result in compression injuries and avulsion disruption of soft tissues. Inadequate post-injury management often leads to dysfunction or even loss of function, resulting in a high rate of disability (26). The treatment process sometimes requires multidisciplinary teamwork. Certain chemicals, such as hydrofluoric acid, are highly toxic and can cause systemic toxicity or life-threatening injuries even with small doses (27). Diagnosis of electrical burn wounds is generally not difficult, but it is crucial to remain vigilant for signs of deep tissue involvement and insidious visceral injuries during treatment (28).

In this study, the mortality rate of older adult patients aged ≥60 years old was 1.87%, which is lower than previous studies (15, 29, 30). In patients with explosion injuries and TBSA area > 50%, the mortality rate was statistically higher. We should pay more concern to the vital signs of such patients and to allocate medical resources in a rational manner during the treatment of burns. The rate of surgery in patients decreased with increasing age. This may be because younger patients have a more positive attitude toward burn treatment. Older adult patients have difficulty tolerating surgery due to economic or physical reasons, and some may opt for conservative treatment or leave the hospital against medical advice (31). The factors associated with hospitalization costs in this study included age, TBSA, number of surgeries and length of hospitalization. Compared to scalding and flame injuries, explosion injuries, hot crush injuries, and electric burns have longer LOS and higher hospitalization costs. This places a greater financial burden on the patient’s family. In addition to preventing the occurrence of such accidents, there is a need to make clinical decisions about treatment, care, and rehabilitation according to the patient’s age, TBSA, the cause of the injury. The reasonable arrangement of medical information can reduce unnecessary surgery and expenses, alleviate patients’ financial pressure, and help them recover early and integrate into society and family life.

When analyzing the results of this study, the following limitations must be considered. Firstly, the burn center included in this study is the primary burn center in the Suzhou region. Data from other hospitals in the region were not included, which may make our results unrepresentative of the eastern region of China. Additionally, outpatients and emergency patients were excluded in this study, whose burn severity was generally lower than inpatients.

## Conclusion

The analysis of data at different stages can provide new guidance and references for the development of prevention measures. The results of this study suggest that education and protective measures for workplace safety should be strengthened for middle-aged men. To prevent occupational injuries, different prevention strategies should be developed according to different occupational characteristics. For the female and older adult population, there should be a formal publicity campaign on common risk factors in life, prevention, and pre-hospital first aid treatment. In clinical practice, it is imperative that burn treatment and rehabilitation strategies be developed in a reasonable, adequate, and individualized manner, taking into account the patient’s age, etiology, and degree of burn injury.

## Data availability statement

The raw data supporting the conclusions of this article will be made available by the authors, without undue reservation.

## Ethics statement

The studies involving humans were approved by the Ethics Committee of the Affiliated Suzhou Hospital of Nanjing Medical University, Suzhou Municipal Hospital. The studies were conducted in accordance with the local legislation and institutional requirements. The participants provided their written informed consent to participate in this study.

## Author contributions

YZ: Conceptualization, Data curation, Investigation, Visualization, Writing – original draft, Writing – review & editing. JS: Funding acquisition, Investigation, Resources, Supervision, Writing – review & editing. YL: Formal analysis, Investigation, Validation, Visualization, Writing – review & editing. RaS: Formal analysis, Funding acquisition, Investigation, Validation, Writing – review & editing. RuS: Conceptualization, Project administration, Supervision, Visualization, Writing – original draft, Writing – review & editing.
